# Further exploration during open appendicectomy; assessment of some common intraoperative findings

**Published:** 2014

**Authors:** Adetunji Saliu Oguntola, Moses Layiwola Adeoti, Sulaiman Olayide Agodirin, Adetunji Adeniyi Oremakinde, Kunle O Ojemakinde

**Affiliations:** 1 Adetunji Saliu Oguntola, Department of Surgery, Lautech Teaching Hospital, Osogbo, Nigeria.; 2 Moses Layiwola Adeoti, Department of Surgery, Lautech Teaching Hospital, Osogbo, Nigeria.; 3 Sulaiman Olayide Agodirin, Department of Surgery, Lautech Teaching Hospital, Osogbo, Nigeria.; 4 Adetunji Adeniyi Oremakinde, Department of Surgery, Lautech Teaching Hospital, Osogbo, Nigeria.; 5 Kunle O Ojemakinde, Department of Histopathology, Lautech Teaching Hospital, Osogbo, Nigeria.

**Keywords:** Appendicitis, Exploration, Greater omentum, Histology, Macroscopic appearance, Peritoneal exudates

## Abstract

***Objective: ***Very few studies are available to relate the final histology of excised appendix with the detailed intra-operative findings during appendectomy, both open and laparoscopic. This study was aimed to correlate the histological features of appendix specimen with the intra operative findings at open appendicectomy (OA) in a bid to determine when to change the planned procedure to include further exploration.

***Methods*** ***:*** A prospective study that observes the condition of the greater omentum (GO), the vermiform appendix and peritoneal exudates at all OA done for uncomplicated appendicitis. Histological examination of the appendices done using the H&E stain.

***Results*** ***: ***Eighty-five patients had emergency open OA, their^’^ ages range from 6 to 62 yrs (median = 23yrs). Histology showed 7 normal appendix (HNA), 56 acute (HAA) and 22 “non acute” appendicitis (HNAA). Negative appendicectomy rate was 8.2%. The GO was sighted more in patients with HAA than HNAA (p=0.00015) and also significantly more inflamed in the former (p=0.00028). It is not significantly inflamed in those with HNAA (p=0.945). The negative predictive value (NPV) of absent GO is 35.7% while the positive predictive value (PPV) of sighted normal GO and inflamed GO are 92.8% and 100% respectively. The PPV and NPV of presence of pus for diseased appendix are 95.8% and 9.8% respectively while those of excess fluid are 94.8% and 10.8%. The PPV and NPV of macroscopic assessment of the appendix for inflammation are 97% and 45.5% respectively giving the diagnostic accuracy of 90.6%. A significant trend of increasing probability of histologically inflamed appendix with increasing severity of macroscopic feature was seen (X^2^ = 004 df=1, p<0.005).

***Conclusion:*** High positive and low negative predictive values are similar for all the three parameters assessed. The macroscopic appearance of the appendix has a predictive likelihood ratio for further exploration.

## INTRODUCTION

It is very distressing for both the surgeon and the patient when an operation has been performed and the symptoms do not resolve, it leads to continuing morbidity and sometime loss of trust between the patient and the clinician. This is a likely scenario when an appendectomy is wrongly performed in place of one of the many close differentials that may mimic it.^1^^-^^3^ Appendicitis, the most common indication for emergency operation in general surgical services worldwide has numerous differentials, yet the decision to operate is usually based on clinical signs and symptoms which are not fool proof ^3^^,^^4^ and on some occasions supported with ancillary diagnostic support like abdominal ultrasound, especially in children and females of child-bearing age.^1^^,^^4^

If the decision has been made to operate on a case of suspected uncomplicated appendicitis, most of the time, the appendix is approached through a small right iliac fossa muscle splitting incision^4^, and the appendix is removed without further explorations. If however the appendix is suspected to be normal, the surgeon may have an opportunity to make the right diagnosis and perhaps treat appropriately by continuing with a mini exploration for the close differentials, a procedure which may be cost effective if appropriately instituted but with its own associated problems.

The intra-operative findings, like the gross appearance of the appendix, the presence and appearance of the omentum and the presence and quality of peri-ceacal collection help in making a decision whether or not to conduct a mini-exploration hence reduce the rate of failure to resolve the patients symptoms after a negative appendectomy.

Though the gold-standard for diagnosis of appendicitis is histology^5^ the observatory ability of the surgeon plays a major role in determining modification of the planned procedure, a decision which makes a difference in patient satisfaction, the associated postoperative morbidity, operation time and surgical outcome.

When the appendix is inflamed, there is exudation of fluid from the reaction of the peritoneum, the fluid may be initially serous, then turbid and later purulent as the inflammation progresses or with advent of complications. The greater omentum (GO) once described as the “policeman of the abdomen” moves towards the right iliac fossa region in an attempt to wall off the inflamed appendix thus limiting the spread of the toxic inflammatory fluid in the peritoneal cavity. The use of this theoretical knowledge of the proceedings in the peritoneal cavity is invaluable. This study compares the histological features of appendix specimen, with the intra operative findings at open appendicectomy (OA) for clinically suspected appendicitis at LAUTECH Teaching hospital (LTH) between January 2007 and December 2008 with the aim of proposing when to suspect negative appendectomy intra-operatively and consider modification of the planned procedure.

## METHODS

This is a prospective study of all open emergency appendectomies done for clinical diagnosis of uncomplicated appendicitis over a two year period in the general surgery division of the department of surgery LTH Osogbo, Nigeria. All patients were diagnosed by the team under the leadership of at least one of the consultant General surgeons who takes decision on surgical intervention. Exclusion criteria include features of extensive localised peritonitis, appendicial mass and those who had interval appendectomy. The operations were done through a Lanz’s incision by a team of a Senior Registrar and a Registrar. The location of the omentum (present or not around the appendix) and its gross appearance were noted (thickenend / indurated or edematous). The gross appearance of the appendix, presence of serous or purulent fluid in the right iliac fossa were noted, seeping out of serous peritoneal fluid upon opening the anterior parietal peritoneum or at mobilization of the ceacum or appendix was considered presence of excess peritoneal fluid . Presence of pus was noted irrespective of the volume. The degree of inflammation of the appendix was subjectively graded as follows; Sub-serosal vascular engorgement as mild inflammation, fibrinous exudates on the appendix and edematous appendix as moderate inflammation and necrotic/ gangrenous as severe inflammation. Presence of complications like perforation or rupture was also noted. Histological diagnoses made by Haematoxylin and Eosin stain were done for all reported cases by the histopathologists.

The operative findings of histologically normal appendix (HNA) were compared with those of the histologically pathologic appendix. Appendix that were not acutely inflamed were grouped together as histologically non acute appendicitis (HNAA), while those that were acutely inflamed were referred to as Histologically acute appendicitis (HAA). Results are presented in descriptive statistics. Comparison was made using students t-test and chi-square. P-value of less than 0.05 was considered significant. The likelihood-ratio was calculated for all the parameters.

## RESULTS

Eighty five of all the 92 patients who had open emergency appendicectomy for clinically diagnosed appendicitis during the study period were included (histology report of 7 were not available). Fifty three percent (n= 45) were males. Ages ranged from 6 to 62 yrs (median age= 23 yrs). Eight percent (n=7) of the removed appendices were normal histologically. (Table-1) The greater omentum (GO) was more frequently seen in the right iliac fossa in patients with HAA (n=16) than in those with HNAA (n=10), (p=0.00015) (Table-II), also thickening or edema of the omentum was more common in patients with HAA (n=38) than in those with HNAA (n=5), (p=0.00028). The NPV of absence of GO in the right iliac fossa was 35.7% while the PPV of sighted normal GO and sighted inflamed GO are 92.8% and 100% respectively.

Excess peritoneal fluid was present in 39 (46% of all patients). Seventy-four percent (n=29) of these are in patients with HAA. About fifty-two percent (29 of 56) and 36.4% (8 of 22) of HAA and HNAA respectively had excess peritoneal fluid. Pus was present in a total of 24 patients (28.2%), 79% (n=19) of whom had HAA. Nineteen out of 56 (33.9%) of HAA, 4 of 22 (18.2%) of HNAA and one of 7 HNA had pus around the appendix (Table-III). The PPV and NPV of pus for disease appendix are 95.8% and 9.8% respectively while those of excess fluid are 94.8% and 10.8%.

Eleven appendices appear macroscopically normal overall. None of those with histological diagnosis of schistosomal appendicitis, lymphoid hyperplasia and atrophic appendicitis appeared normal macroscopically. Two of the 68 appendix that look inflamed were found to be histologically normal (false positive rate of 2.9%). Six out of 11 thought to be normal were inflamed histologically (false negative 54.5%) The PPV and NPV of macroscopic assessment of the appendix for inflammation are 97% and 45.5% respectively giving the diagnostic accuracy of 90.6% see Table-IV. Six clinically unsuspected perforation or rupture were encountered intra-operatively (Table-IV).

Seventy-six and half percent (52 of 68) of the macroscopically inflamed appendices were found to be HAA (92.3% of these (48/52) were described as moderate to severe inflammation). Forty percent, 83% and 80% of the macroscopically mild (4 /10), moderate (40/48) and severely (8 /10) inflamed appendix respectively came out as HAA while 50% (5/10), 14.6% (7/48), and 20% (2/10) respectively as well are reported as HNAA (Table-IV).

Analysis done using Chi-square test for trend to check for linear trend in proportion for appendix adjudged to be mildly, moderately or severely inflamed using corresponding histological grading as the gold standard showed a significant trend of increasing probability of histologically inflamed appendix with increasing severity of macroscopic feature. (X^2^ = 004 df=1, p<0.005. Mild, moderate and severe scored as 1, 2, and 3 respectively). The likelihood ratio of each of the observed intra-operative signs is as shown in Table-V.

## DISCUSSION

Surgeons continue to meet challenges in the diagnosis of appendicitis world-wide. The introduction of various scoring methods, intra-operative assessments^6^, frozen section^5^, radiological investigations^7^ and laparoscopic procedure to reduce the rate of negative appendectomy have all been met with varying results.^1^^,^^3^^,^^5^^-^^7^

**Table-I:** Histologic finding against presence of inflamed/edematous greater omentum

*Histology*	*Inflamed*	*Seen but not inflamed*	*Not Seen*	*Total*
Normal	0	2	5	7 (8.2%)
Acute Suppurative/ Ulcerative Necrotic	38	16	2	56 (65.99%)
Schisto Somal	1	1	1	3 (3.5%)
Atrophic	1	0	1	2 (2.4%)
Chronic Non Specific Appendicitis	0	2	2	4 (4.7%)
Lmphoid Hyperplasia	1	2	0	3 (3.5%)
Submucosa Fibrosis	2	5	3	10 (11.8%)
Total	43	28	14	85 (100%)

**Tables-II:** Presence of PUS and excess peritoneal fluid against histological diagnosis

	*PUS*		*Excess Peritoneal Fluid*		
*Histology*	*Present*	*Absent*	*Present*	*Absent*	*Total*
Normal	1	6	2	5	7
Acute Suppurative/ Ulcerative Necrotic	19	37	29	27	56
Schisto Somal	1	2	1	2	3
Atrophic	0	2	1	1	2
Chronic Non Specific Appendicitis	0	4	1	3	4
Lmphoid Hyperplasia	1	2	2	1	3
Submucosa Fibrosis	2	8	3	7	10
Total	24	61	39	46	85

**Table-III:** Histologic finding against the macroscopic observation of the appendix

*Histology*	*Normal*	*Mildly Inflamed*	*Moderately Inflamed*	*Severely inflamed*	*Subtotal inflamed*	*Perforated/ Ruptured*	*Total*
Normal	5	1	1	0	2	0	7
Acute Suppurative/ Ulcerative Necrotic	2	4	40	8	52	2	56
Schisto Somal	0	1	1	0	2	1	3
Atrophic	0	0	1	0	1	1	2
Chronic Non Specific Appendicitis	2	1	0	0	1	1	4
Lmphoid Hyperplasia	0	1	0	1	2	1	3
Submucosa Fibrosis	2	2	5	1	8	0	10
Total	11	10	48	10	68	6	85

**Table-IV:** Likelihood for exploration for each appendectomy findings

	*Likelihood Ratio (LR)*	
	*Positive*	*Negative*
Macroscopically inflamed appendix	3.2	0.1
Excess peritoneal fluid or presence of pus in the right iliac fossa	3.1	0.8
Presence of omentum in the right iliac fossa	1.1	0.86

Jones AE et al^5^ concluded that intra-operative diagnosis of the surgeon is unreliable in detecting abnormality of the appendix, but this was challenged by Kraemer M et al^8^ when he reported a false negative rate of 3% with laparoscopic appendicectomy (LA). The location of the GO around the site of inflammation is a rather efficient physiologic response to inflammation. In this study the presence of edematous or thickened appendix was unequivocal for acute appendicitis. Unfortunately the absence of GO did not sufficiently predict that the appendix is normal, hence it may not be used to decide whether to proceed with further exploration The finding of excess peritoneal fluid or pus in the HAA group compared to HNAA is expected because acute inflammatory reactions elicit more rapid exudation and irritation than the chronic types. A subgroup of appendicitis due to parasitic infestation e.g. Schistosomiasis, oxyuriasis, enterobiasis, atrophic appendicitis, sub-mucosal fibrosis and rare findings like endometriosis^9^ are unlikely to stimulate intense inflammatory reactions.

In this study, excess clear peritoneal fluid was more common than pus. This is an expected finding because clinically complicated cases were excluded. The time from onset of symptom to presentation was not included, but it may be assumed that most cases presented early because later presentation tend to have more complications and pus collection.

The NA rate in open appendectomy (OA) is known to be less than that of LA.^8^ Positive and negative predictive values of 97% and 45% found in this study is similar to that of Cheuk et al^10^ from OA from Singapore, Kreamer et al^8^ and Chiarugi et al^11^ from OA (Italy). The presence of a senior registrar with at least 3-4 years of surgical experience in the operating room will not only contribute to this but also help in reducing the risk of leaving behind a second pathology. The PPV and NPV of LA procedure has been improved by the ZM signal method of transillumination pulsomotorographic monitoring of functional characterization of the vermiform appendix and caecum during operation.^12^

The presence of inter-observer error among surgeons and very importantly the presence of inflammation only in the mucosa and submucosa in early appendicitis (endo-appendicitis), in addition to the presence of some form of subacute appendicitis which may not have macroscopic signs of inflammation will all contribute to the high false negative rate when diagnosis is based on operative findings. Per-operative inspection of the appendix mucosa aimed at improving the diagnostic accuracy has not been effective, and it may also compromise the subsequent histological examination.^6^ Hence it was not considered in this study.

Presently, histology is the gold-standard for confirmation of appendicitis, but, a subgroup of inflamed appendix may be histologically normal but with molecular evidence of inflammatory changes. In a study, this was found in up to about 50% of histologically normal appendix from patients with clinical diagnosis of appendicitis.^13^ This subgroup also contribute to the high rate of negative appendectomy.

A major essence of assessing the macroscopic appearance of the appendix is to determine the need for further exploration when the appendix appears normal, in order to explore for differentials such as appendicitis, Meckel’s diverticulitis, regional enteritis, diverticular disease of the colon and right tubo-ovarian lesions in females through the same right iliac fossa incision. If unnecessary exploration is done, it adds to operation time, may cause spread of contamination, could possibly delay return of bowel activity; all these could heighten the post-operative morbidity. Hence it is not advisable to engage in routine exploration once the appendix is found to be macroscopically inflamed.

It should be remembered that in the absence of an inflamed appendix, the presence of other signs; GO in the right iliac fossa, excess peritoneal fluid or pus may be from other pathologies in the right iliac fossa. Where possible, Laparoscopy appendectomy is known to be safer and with lower complication rate^14^ and can easily be used to explore thus minimizing contamination and missed pathology.^4^

Non-uniformity in the intraoperative classification or description of the appendix worsened by poor agreement among surgeons in describing the severity of appendicitis as found by Ponsky TA et al contributed to the inconsistency in the assessment of outcome of comparison.^15^ The macroscopic grading of the severity of the inflammation of the appendix in this study showed increasing accuracy with the progression of the grade. Though the staging method is subjective, a consensus on grading the degree of inflammation of the appendix will be helpful. Kell MR et al^16^ described a simple grading system, the Intra-operative Appendicitis Severity Score (IASS), this can be modified for a general acceptance, probably to include parameters like the state of the greater omentum, presence or absence of pus or excess fluid and grade of inflammation of the vermiform appendix (if found to be inflamed). This will create uniformity, reduce inter-observer variation and make observation less subjective. It will also create grounds room for easy comparison of results.

The likelihood ratios of each of the intra-operative findings suggest that in the absence of inflamed appendix one may progress to exploration. However the same may not be said of the other intra-operative signs used in this study. One factor that may improve the specificity or sensitivity of these signs is sex segregation, where the signs are used for each sex separately rather than combined analysis for both sexes because females tend to have more differentials in relation to the reproductive tract.^3^

## CONCLUSION

Making a correct on-table assessment that an appendix is normal is still a challenge to surgeons worldwide in view of the high false negative rate. Similar high positive and low negative predictive values are obtained for presence of the greater omentum in the RIF region, macroscopic appearance of the appendix and presence of pus or excess peritoneal fluid in this study. The likelihood ratios of each of the intra-operative findings confirms that it is only in the absence of inflamed appendix one may progress to exploration. The diagnostic accuracy can be improved by developing a standard scoring scale comprising of the state of the above assessed parameters.

## Authors contributions:

**Dr. AS Oguntola: **Study concept, study design, Literature search and write up.

**Dr. ML Adeoti: **Study design, literature search and final write up.

**Dr SO Agodirin: **Literature search and statistical analysis.

**Dr AA Oremakinde: **Collection and collation of data, literature search.

**Dr. KO Ojemakinde: **Literature search and Histopathology.

## Notes

***Source of Funding:*** Personal Contributions.
